# Media use and sleep in twins, an important new dimension of a causal puzzle

**DOI:** 10.1093/sleep/zsad042

**Published:** 2023-04-28

**Authors:** Jan Van den Bulck

**Affiliations:** Department of Communication and Media, University of Michigan, Ann Arbor, MI, USA

The past few decades have seen meteoric growth in the number of media and in the types of content they provide. In addition to that, media devices have become more and more portable. We now have access to news, entertainment, and interaction modes on mobile phones, tablet computers, and laptops that allow us to expose ourselves to these media wherever we like, especially since the advent of wireless internet and mobile phone technology. Media follow us around. While media used to have dedicated places where we kept a television or radio set, we now have access to them whenever and wherever we like, even at work or, more importantly in the context of this paper, in the bedroom [1].

The rise in media availability and use has coincided with another remarkable trend. It has been estimated that people now sleep 1 to 2 hours less than they did half a century ago [2, 3]. Around the turn of the century, researchers started to wonder whether media use and sleep were related [4], a concern that became more urgent once the mobile phone started to make people continuously accessible to others and, more importantly, increasingly offered that same option to children. A study of 2546 adolescents published in 2003 found that while adults were still adjusting to the new technology, teenagers communicated with each other at alarming numbers until deep into the night [5].

By 2015, there was so much research on the relationship between media use and sleep issues that Hale et al. could comprehensively review 67 studies on the topic [6]. Media use was found to coincide with several sleep indicators, including sleep duration, sleep latency, and sleep quality.

Although it is fair to claim that the relationship between media use and sleep is now well established, explaining the mechanisms behind the connection has been much more difficult. Media use has been described as “a residual category of leisure activity.” [7] It is habit driven and often occurs without much thought. This presents two big research problems. First, in the context of sleep laboratories, where sleep indicators can be monitored accurately via polysomnography, those habitual behaviors are unlikely to resemble what happens in a natural setting. To put it bluntly, hooked up to dozens of sensors, monitored by technicians and cameras, few people are likely to exhibit the behaviors that come naturally at home, such as binge viewing [8] or social media scrolling [9]. Second, in nonclinical or nonexperimental settings, researchers must rely on self-reports for both the media and the sleep measures. This provides a double dose of measurement bias and recollection issues.

Despite these hurdles, there is now so much research that several mechanisms have been put forward to explain how media use is likely to affect sleep.

The first dimension that has been identified is a physiological one. The topic that has received the most attention in this field is the effect of shortwave or blue light on the sleep–wake cycle [10–12]. Many modern media devices emit such blue light, and newer developments seem to coincide with behaviors that put the screens (and therefore the light source) of these devices closer to the eyes of the user. Using a clever study design, Chang et al. thus showed that reading a paper book has a different effect than reading the same content on a so-called e-reader [13].

The second dimension is best described as a psychological one. Good examples of this approach are studies looking at the arousal effects of media use. Children who have been exposed to media content that is targeted at adults have been shown to have a higher risk of sleep problems [14]. When parents were advised to replace violent media content with more positive messages, young children were found to show improved sleep outcomes [15]. Disturbing media content has also been shown to coincide with nightmares [16].

In this issue of *SLEEP,* Madrid-Valero et al. introduce an important new dimension of the relationship between media use and sleep [17]. Individual reactions (or the lack of a reaction) to media use are so varied that they were described as early as 1948 as: “some kinds of communication on some kinds of issues, brought to the attention of some kinds of people under certain kinds of conditions have some kinds of effects.” [18] Despite the age of that observation, research on genetic predispositions to media use and media effects is exceedingly rare [19]. It begs the question of whether any relationship between media use and sleep might differ for each individual and whether genetics is the missing link in this puzzle. Perhaps a previously unknown factor creates a spurious link by influencing both behaviors. The study of Madrid-Valero et al. is, therefore, unique and important.

The authors conducted a study of an impressive 2232 18-year-old twins. The use of monozygotic and dizygotic twins, combined with a longitudinal design spanning almost a decade and a half, provides remarkable data that allowed the authors to conduct an unparalleled test of genetic and environmental factors. This elegant study suggests that genetics certainly play a role but cannot explain everything that goes on in the relationship between media use and problematic sleep outcomes.

This study is not the final word. Other dimensions could be studied more closely. In addition to the familial factors cleverly modeled by Madrid-Valero et al., sociological variables like employment status, financial insecurity, and racial or ethnic discrimination deserve more attention. The media provide an additional dimension. Streaming technology cleverly encourages binge viewing, and push messages on mobile phones reinforce social media habits. Still, the genetic component of the puzzle is such an important factor that this study can undoubtedly be referred to as pivotal.

The relationship between media use is complex. Describing causal mechanisms requires sophistication. Inevitably, all future technological developments will only lead to more media, content, and ways to fill more parts of our daily lives with them. Slater described media effects using the metaphor of “reinforcing spirals,” where one element reinforces all the others continuously [20]. Media use can lead to sleep issues, which can influence our experience of basic needs, or our levels of self-control, which affect our media use. Thanks to Madrid-Valero colleague al., we have identified an essential new spiral to examine further.

## References

[1] Bovill M , et al. Bedroom culture and the privatization of media use. In: Livingstone SM, Bovill M, eds. Children and their Changing Media Environment: A European Comparative Study. Mahwah, N.J.: L.Erlbaum Associates, 2001: 179–200.

[2] Bixler E. Sleep and society: an epidemiological perspective. Sleep Med. 2009;10(supp 1):S3–S6. doi: 10.1016/j.sleep.2009.07.005 19660985

[3] Cappuccio FP , et al. Is prolonged lack of sleep associated with obesity? BMJ. 2011;342:10–12. doi: 10.1136/bmj.d3306 21622519

[4] Van den Bulck J. Is television bad for your health? behavior and body image of the adolescent “Couch Potato.”. J Youth Adoles. 2000;29(3):273–288.

[5] Van den Bulck J. Text messaging as a cause of sleep interruption in adolescents, evidence from a cross-sectional study. J Sleep Res. 2003;12(3):263–263. doi: 10.1046/j.1365-2869.2003.00362.x 12941066

[6] Hale L , et al. Screen time and sleep among school-aged children and adolescents: a systematic literature review. Sleep Med Rev. 2015;21:50–58. doi: 10.1016/j.smrv.2014.07.007 25193149 PMC4437561

[7] McQuail D , et al. The television audience: a revised perspective. In: McQuail D, ed. Sociology of mass communications: selected readings. Harmondsworth: Penguin; 1972.

[8] Exelmans L , et al. Binge viewing, sleep, and the role of pre-sleep arousal. J Clin Sleep Med. 2017;13(8):1001–1008. doi: 10.5664/jcsm.6704 28728618 PMC5529125

[9] Bergfeld NS , et al. It’s not all about the likes: Social media affordances with nighttime, problematic, and adverse use as predictors of adolescent sleep indicators. Sleep Health. 2021;7(5):548–555. doi: 10.1016/j.sleh.2021.05.009 34281814

[10] Wood B , et al. Light level and duration of exposure determine the impact of self-luminous tablets on melatonin suppression. Appl Ergon. 2012;44(2):237–240. 22850476 10.1016/j.apergo.2012.07.008

[11] Cajochen C , et al. Evening exposure to a light emitting diodes (LED)-backlit computer screen affects circadian physiology and cognitive performance. J Appl Physiol. 2011;110(5):1432–1438. doi: 10.1152/japplphysiol.00165.2011 21415172

[12] Berson DM , et al. Phototransduction by retinal ganglion cells that set the circadian clock. Science. 2002;295(1998):1070–1073. doi: 10.1126/science.1067262 11834835

[13] Chang A , et al. Evening use of light-emitting ereaders negatively affects sleep, circadian timing, and next-morning alertness. Proc Natl Acad Sci. 2015;112(4):1232–1237. 25535358 10.1073/pnas.1418490112 PMC4313820

[14] Paavonen EJ , et al. TV exposure associated with sleep disturbances in 5 - to 6-year-old children. J Sleep Res. 2006;15(2):154–161. doi: 10.1111/j.1365-2869.2006.00525.x 16704570

[15] Garrison MM , et al. The impact of a healthy media use intervention on sleep in preschool children. Pediatrics. 2012;130(3):492–499. doi: 10.1542/peds.2011-3153 22869826 PMC3428755

[16] Van den Bulck J , et al. (2016). Violence, sex, and dreams: violent and sexual media content infiltrate our dreams at night. Dreaming. 2016;26(4):271–279. doi: 10.1037/drm0000036

[17] Madrid-Valero J , et al. Problematic technology use and sleep quality in young adulthood: Novel insights from a nationally representative twin study. Sleep. 2023;46(6):zsad038. 10.1093/sleep/zsad038 PMC10262182 37106487

[18] Berelson, B. Communications and public opinion. In W. Schramm (Ed), Communications in modern society (pp. 167-185). Urbana, IL: University of Illinois Press; 1948.

[19] Nikkelen SW , et al. Media violence and children’s ADHD-related behaviors: a genetic susceptibility perspective. J Commun. 2014;64(1):42–60.

[20] Slater MD. Reinforcing spirals: the mutual influence of media selectivity and media effects and their impact on individual behavior and social identity. Commun Theory. 2007;17(3):281–303. doi: 10.1111/j.1468-2885.2007.00296.x

